# Deciphering the Molecular Basis for Attenuation of *Flavobacterium columnare* Strain Fc1723 Used as Modified Live Vaccine against Columnaris Disease

**DOI:** 10.3390/vaccines9111370

**Published:** 2021-11-22

**Authors:** Wenlong Cai, Covadonga R. Arias

**Affiliations:** 1 Department of Infectious Diseases and Public Health, Jockey Club College of Veterinary Medicine and Life Sciences, City University of Hong Kong, Kowloon, Hong Kong 999077, China; 2 School of Fisheries, Aquaculture and Aquatic Sciences, Auburn University, Auburn, AL 36832, USA; ariascr@auburn.edu

**Keywords:** *Flavobacterium columnare*, columnaris disease, attenuated vaccine, rifampin, catfish

## Abstract

Vaccines are widely employed in aquaculture to prevent bacterial infections, but their use by the U.S. catfish industry is very limited. One of the main diseases affecting catfish aquaculture is columnaris disease, caused by the bacterial pathogen *Flavobacterium columnare*. In 2011, a modified-live vaccine against columnaris disease was developed by selecting mutants that were resistant to rifampin. The previous study has suggested that this vaccine is stable, safe, and effective, but the mechanisms that resulted in attenuation remained uncharacterized. To understand the molecular basis for attenuation, a comparative genomic analysis was conducted to identify specific point mutations. The PacBio RS long-read sequencing platform was used to obtain draft genomes of the mutant attenuated strain (Fc1723) and the parent virulent strain (FcB27). Sequence-based genome comparison identified 16 single nucleotide polymorphisms (SNP) unique to the mutant. Genes that contained mutations were involved in rifampin resistance, gliding motility, DNA transcription, toxin secretion, and extracellular protease synthesis. The results also found that the vaccine strain formed biofilm at a significantly lower rate than the parent strain. These observations suggested that the rifampin-resistant phenotype and the associated attenuation of the vaccine strain result from the altered activity of RNA polymerase (RpoB) and possible disrupted protein secretion systems.

## 1. Introduction

*Flavobacterium columnare* is the causative agent of columnaris disease, which can infect a broad range of fish hosts, including wild, cultured and ornamental species [1]. In the United States, *F. columnare* is one of the leading pathogens affecting the catfish industry, with in-pond mortality rates reaching up to 60 and 90% in adults and fingerlings, respectively [2]. *F. columnare* is a ubiquitous aquatic bacterium [3] that can form biofilm [4] and survive in water for up to 6 months [5]. Meta-genomic analysis of *F. columnare* revealed that the species is capable of denitrification, which could allow for anaerobic growth in pond sediments [6].

Since the eradication of *F. columnare* from aquaculture systems is very unlikely, prevention practices are the best approach to reduce the incidence of columnaris disease in farms. It is well known that the use of vaccines in fish farms contributes to more sustainable production systems by eliminating or reducing antibiotic use [7]. Among the several types of vaccines available, live attenuated vaccines elicit a stronger immune response and a long-lasting humoral and cell-mediated protection than bacterins [8,9]. Currently, there is a commercial, live attenuated vaccine against columnaris disease under the trade name AQUAVAC-COL^TM^ (Merck Animal Health, Madison, NJ, USA). However, its efficacy has been questioned, and it is not widely used by farmers [10].

*F. columnare* can be divided into several genetic groups, or genomovars, based on the 16S rRNA gene sequence [11,12]. Genomovar II strains exhibited host-specificity toward catfishes in aquatic environments [13] and a higher degree of virulence towards channel catfish than genomovar I isolates [10,14]. AQUAVAC-COL^TM^ derives from a genomovar I strain and offers little protection against the most virulent strains of *F. columnare*, not only in channel catfish but also in tilapia and zebrafish [10]. In 2011, A new attenuated vaccine was developed derived from a highly virulent genomovar II strain of *F. columnare* using a rifampin-resistant selection strategy [15]. The previous study suggested that the vaccine is stable, safe, and effective, but the mechanisms that resulted in attenuation remained uncharacterized [10]. In this study, comparative genomics analysis was performed to identify the point mutations that occurred in the rifampin-resistant mutant and may have resulted in the attenuated phenotype.

## 2. Materials and Methods

### 2.1. Bacterial Strains and Growth Conditions

*F. columnare* strain FcB27 was isolated from channel catfish in Alabama in 2005 [13], and it caused a cumulative mortality of 53.0 ± 8.6% in channel catfish fingerlings in our previous study [10]. The mutant strain Fc1723 was obtained by our group by passing strain FcB27 on an increasing concentration of rifampin (for a detailed description see Olivares-Fuster et al., [15]), and it provided protection against control groups by a relative percentage survival (RPS) of 54.7% under the laboratory condition [10]. Bacteria were grown at 28 °C in Modified Shieh (M.S.) broth medium for 48 h with shaking at 200 rpm [16]. Stock suspensions of the isolates were stored in M.S. broth with 20% glycerol at −80 °C.

### 2.2. Genomic DNA Extraction

Bacterial DNA was extracted using a Qiagen DNeasy Blood & Tissue kit (Qiagen, MD, USA) following the manufacturer’s instructions, including the RNase incubation step. All the DNA samples were quantified using a NanoDrop ND-1000 Spectrophotometer (Thermo Scientific, Wilmington, DE, USA) and separated on a 1% agarose gel for integrity check.

### 2.3. Genomic Sequencing and Assembly

The genomic library was prepared using a DNA Template Prep Kit 1.0 (PacBio) with a 20 kb preparation protocol. Afterwards, the sequencing was performed with the PacBio long-read sequencing RS II P6-C4 chemistry platform. PacBio subreads were filtered with default parameters, and a total of 1.55 Gb and 1.28 Gb of subreads were generated from parent strain FcB27 and mutant strain Fc1723, respectively. In general, strain FcB27 yielded 534,427 subreads with an average length of 3064 bp, while strain Fc1723 generated 418,658 subreads with an average length of 3579 bp. Genome assembly was performed using PacBio PBcR HGAP 2.3 pipeline using de novo assembly protocol under the default settings, and contigs with mean quality values (QV) of less than 45 were filtered out. In the end, four contigs with a total length of 3.45 Mb (mean coverage = 281×, mean QV = 48.63) were obtained from strain Fc1723, and 6 contigs with a total length of 3.45 Mb (mean coverage = 281×, mean QV = 48.63) were acquired from strain FcB27. The library preparation and sequencing were carried out at the University of Washington PacBio Sequencing Service center (Seattle, WA, USA). A QV of 45 ensured above 99.997% accuracy [17].

### 2.4. Gene Annotation and SNP Identification

Contig annotation was performed with the Rapid Annotation using Subsystem Technology (RAST) for prokaryotic bacteria [18,19]. Pairwise genome alignment was used to identify the single nucleotide polymorphisms (SNPs) with Mauve version 2.4 [20]. Genes containing mutations were analyzed using BLAST (Altschu et al., 1990), IGV viewer [21], and RAST subsystem database [18]. The functional changes of the amino acid were predicted using PROVEAN Protein software [22].

### 2.5. Biofilm Quantification Assay

Biofilm formation was evaluated as described previously [4]. Briefly, an overnight inoculum was diluted 100 times in M.S. broth, and 100 µL bacterial suspension was added to each well in a 96-well microtiter polystyrene plate (NuncImmuno MaxiSorp; Nunc, Rochester, NY, USA) in quadruplicate. Microtiter plates were incubated for 48 h at 28 °C to allow for bacterial attachment and biofilm formation. General growth was quantified by measuring the optical density (OD) at 595 nm with a MultiSkan FC spectrophotometer (Thermo Scientific, Waltham, MA, USA). Medium containing unattached cells was discarded, and wells were stained with 1% (*w/v*) crystal violet for 20 min. Excess crystal violet solution was removed, and wells were rinsed 3 more times with distilled water. Biofilm formation was quantified by measuring OD_595_ after the remaining dye was dissolved in 96% (*v/v*) ethanol solution. OD values were analyzed using SAS software version 9.2 (SAS Institute, Cary, NC, USA). The significant difference was set at *p* ≤ 0.05.

## 3. Results

### 3.1. Draft Genome of F. columnare B27 and 1723

FcB27 and Fc1723 genomes were sequenced using a PacBio RS II platform. In general. a total of 534,427 and 418,658 subreads were obtained for FcB27 and Fc1723, respectively. Contigs of each genome were generated with Hierarchical Genome Assembly Process (HGAP) de novo assembly analysis application. Short-length contigs with a mean QV of less than 45 were filtered out. The remaining contigs were aligned and reordered to construct the draft genomes using Mauve software with *F. columnare* C2 strain genome (GenBank accession number: NZ_CP015107.1) as a reference. In the end, the FcB27 genome was comprised of six contigs with a genome size of 3.43 Mb, and the Fc1723 genome was comprised of four contigs with a genome size of 3.45 Mb. The overall GC content was 31.4% for each genome. RAST identified 3008 and 3061 coding sequences for FcB27 and Fc1723, respectively, which were functionally categorized into 324 and 325 subsystems.

### 3.2. Single-Nucleotide Polymorphisms Identified in Fc1723

Sequence-based genome comparison by Mauve identified 16 SNP unique to the mutant strain Fc1723 (Table 1). Out of those, fourteen resulted in nonsynonymous amino acid (aa) changes and two in a synonymous change (Table 1). Two nonsynonymous mutations were identified in the *rpoB* gene region, which codes for the DNA-directed RNA polymerase β subunit. In *F. columnare*, *rpoB* encodes a protein containing 1341 aa. The *rpoB* gene in the mutant strain Fc1723 presented a double mutation resulting in two aa substitutions: Asn469Lys and His486Tyr substitutions (CDS: AND63316.1 in NCBI). These aa positions (469 and 486 in *F. columnare*) correspond to positions 509 and 526 in the RpoB of *E. coli* K12. There are three clusters within RpoB that have been identified as a rifampin resistance-determining region (RRDR) in *E. coli* (whose *rpoB* encodes for 1342 aa). Cluster I includes aa 507 to 533, cluster II comprises aa 563 to 572, and cluster III corresponds to the single aa 687 [23]. The two *rpoB* mutations identified in Fc1723 fall into the RRDR cluster I region. The PROVEAN (Protein Variation Effect Analyzer) score was −3.43 and −5.73 for the Asn469Lys and His486Tyr substitution, indicating the mutations were deleterious.

There were two mutations that resulted in nonsense codons in a nucleoside permease (NupG) and in a phosphoesterase. The C to A transversion on the minus strand in NupG changed the codon GGA (glysine) to TGA (stop codon), resulting in 240 aa truncated polypeptides from the original 417 aa polypeptides (CDS: AND62982.1 in NCBI). The G to T transversion on the positive strand in the phosphoesterase (annotated as hypothetical protein in NCBI; CDS: AND63983.1) changed the codon GAA (glutamic acid) to TAA (stop codon), resulting in 194 aa truncated polypeptides from the original 333 aa polypeptides.

Five missense mutations resulted in amino acid substitutions in patatin (Phe577Cys), protease IV (Ala470Asp), thiol:disulfide interchange protein (Gly386Arg), thioredoxin reductase (Ile2902Met), and dynein protein (Phe238Ser). There were also two silent mutations identified that resulted in no aa change (protoheme IX farnesyltransferase and dynein heavy chain). Finally, the remaining five mutations observed in Fc1723 occurred in regions of the genome that either encoded hypothetical proteins of unknown function or had not been annotated.

### 3.3. Growth, Gliding Motility, and Biofilm Formation

**Figure 1:**
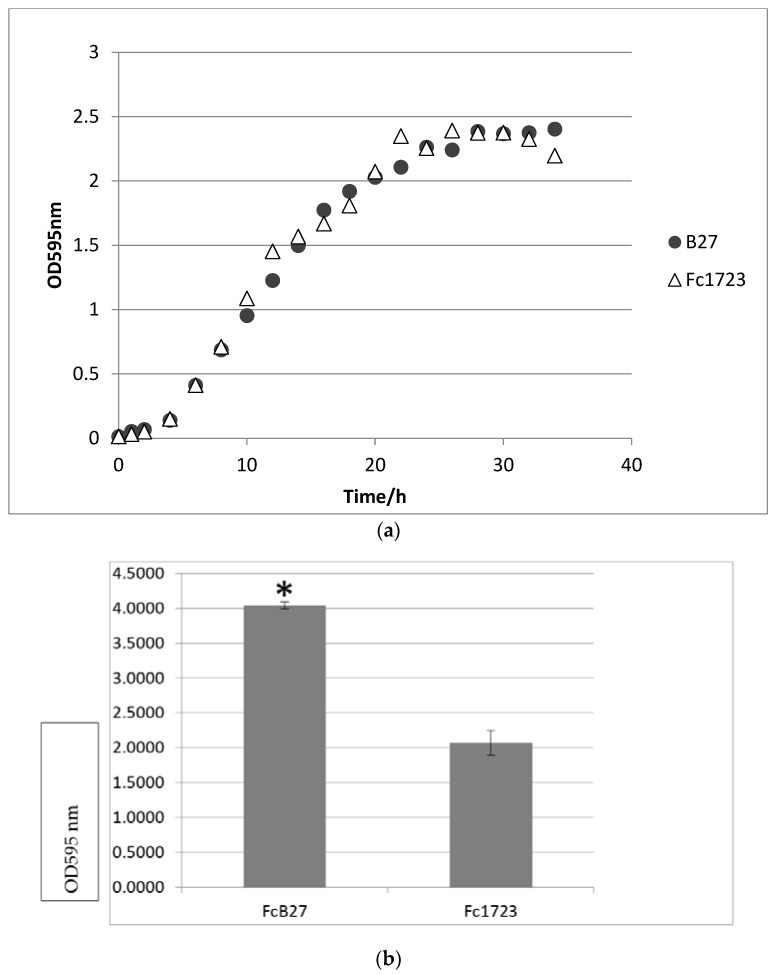
(**a**) Parent strain and vaccine strain growth curves. (**b**) Biofilm formation of virulent parent strain and vaccine strain after 48 h incubation in microtiter plate. * indicates significant difference.

It had been reported that thioredoxin reductase plays an important role in gliding motility and biofilm formation in *F. psychrophilum* [24]. Our result indicated that both parent and mutant strains exhibited gliding motility. No apparent differences were observed between the strains. Since thioredoxin reductase is known for its role in biofilm, biofilm formation between the parent and the mutant strain was further compared. While planktonic growth by both parent and mutant strains was similar (Figure 1a), there was a significant difference in biofilm formation using the microtiter plate method (Figure 1b) between FcB27 and Fc1723. The mutant strain’s ability to form biofilm on microtiter plates was significantly lower than its parent strain.

## 4. Discussion

Attenuated vaccines are regularly used in human and animal medicine to prevent disease. They tend to mimic the route of entry of pathogens, persist longer in the host (can even replicate in some cases) and induce a stronger immune response than bacterins [9,25]. Modified live vaccines elicit a wider range of immunologic responses, including both cell-mediated and humoral, that are generally of greater magnitude and longer duration than those produced by killed or subunit vaccines. In aquaculture, live attenuated vaccines can be easily administered as a timed bath treatment, which significantly reduces vaccine delivery costs when compared with injectable vaccines [26,27]. However, the use of live vaccines has safety concerns associated with the risk of reversion to a virulent form and the possibility of causing disease in immunocompromised individuals [28]. These concerns have hindered the application of live vaccines and, in some cases, have resulted in banning modified live vaccines from use [29,30].

One of the most common strategies to obtain avirulent (attenuated) mutants is by multiple passages of a wild-type strain on increasing concentrations of an antibiotic, for example, rifampin. This process is effective in generating avirulent mutants, but the mechanism(s) responsible for attenuation has remained vastly uncharacterized [31,32,33,34]. Many vaccines had been obtained using this strategy, including *Brucella abortus* (RB51) [32], *Edwardsiella ictaluri* [34], *F. psychrophilum* [33], and *F. columnare* [35]. In this study, the molecular mechanism of a rifampin resistant mutant of *F. columnare* was investigated by identifying the SNPs that differentiated the mutant from its parent strain.

In our study, rifampin resistance was possibly conveyed by two point mutations in the *rpoB* gene in *F. columnare*. Rifampin is a potent, broad-spectrum antibiotic that functions by inhibiting the β-subunit of prokaryotic DNA-dependent RNA polymerase (RNAP) [36]. The molecular basis of rifampin resistance has been extensively studied in *E. coli,* where it has been clearly demonstrated that the drug targets the β subunit of the RNAP encoded by *rpoB* [37]. Comparison of the primary structures of RpoB proteins from different rifampin-resistant bacteria led to the identification of three clusters that contained the majority of the mutations observed in the *rpoB* gene [38]. In Fc1723, two mutations were found within cluster I of RRDR in *rpoB* [23]. It is hypothesized that mutations in the rifampin-resistant RNAP affect the activity of this protein to the extent that protein synthesis is affected. This is supported by some rifampin-resistant mutants exhibiting lower growth rates than their parent strains [31]. However, this was not the case for our mutant Fc1723, which displays a similar growth pattern to its parent strain FcB27. Interestingly, while FcB27 displays the typical rhizoid colonies on the agar plate, Fc1723 presents a smooth colony type [15]. This phenotype could be attributed to the mutations found in *rpoB,* but, more likely, the rifampin-associated attenuation is the result of a synergistic effect of several mutations induced in the course of in vitro passaging. In our previous study, a total of 13 mutants were obtained from different parent strains, and they exhibited different genetic characteristics and virulence [15], which indicated the random mutations genesis during the passage.

Previous studies found that rifampin-resistant attenuated *B. abortus* RB51 and *Edwardsiella ictaluri* displayed altered lipopolysaccharide (LPS) structures that lacked a high molecular mass of LPS fraction. The changes in the LPS structure were considered as the cause for the avirulent phenotype of those mutants [39,40]. In our previous study, however, polyacrylamide gel electrophoresis (SDS-PAGE) of LPS fraction did not show any difference among the LPS profiles between parent strain FcB27 (denoted as AL-CC-17 in [15]) and mutant strain Fc1723. This is consistent with the present genetic analysis, in which no SNPs in genes involved in LPS synthesis were noticed. Similarly, LaFrentz et al. also found that a rifampin-resistant attenuated strain of *F. psychrophilum* did not exhibit different LPS or glycocalyx profiles as compared to the wild-type strain [33]. In addition, Fc1723 exhibited the highest protection rate among all the generated mutants [10]. It is possible that the intact LPS of the mutant strain might trigger a better immunogenic response since it could present an LPS profile same to its parent.

The functionality of a protein is highly dependent on its structure [41]. We identified a nonsense mutation in the gene that codifies for nucleoside permease *nupG*. This transmembrane protein transports purine and pyrimidine across the cell membrane. Nucleobase transport has been studied mostly in *E. coli* and *Bacillus* species [42], where NupG plays a key role in nucleic acid and nucleotide metabolism as it scavenges for exogenous preformed bases to be used in nucleotide biosynthesis [43]. A second nonsense mutation occurred in a gene encoding a phosphoesterase. The mutation at 194 amino acid changed the Glu into a stop codon, resulting in 194 aa premature polypeptides instead of 333 polypeptides (GenBank: AND63983.1). The protein was predicted to be part of the PAP2-like superfamily. Type II phosphatidate phosphatases (PAP2) are transmembrane enzymes, which regulate lipid metabolism by catalyzing the conversion of phosphatidate to diacylglycerol [44]. No correlation between NupG, PAP2, and virulence has been described to date.

Five missense mutations were identified in genes encoding patatin, protease IV, thiol-disulfide interchange protein, thioredoxin reductase, and dynein protein. Patatins are a group of plant storage glycoproteins that show lipid hydrolase activity [45]. The first characterized patatin-like protein (PLP) in bacteria was ExoU in *P. aeruginosa*, which is a type III secreted cytotoxin and a virulent factor with lipase and phospholipase activity [46,47]. Genomes of pathogenic *P. aeruginosa* strains showed a significantly higher number of *plp* genes than those found in genomes of non-pathogenic strains, which suggests a correlation between the *plp* genes and virulence [48]. The authors speculated that a high number of *plp* genes might confer an advantage to the bacterium in its interaction with the host. A thiol-disulfide exchange protein (DsbA) is required for disulfide bond formation in some periplasmic proteins [49]. It facilitates the formation of disulfide bridges and is essential for the correct folding or assembly of many proteins, including toxins, adhesins, and components of the trans-membrane protein secretion system [24]. Since the cytotoxic potential of most common toxins relies on their subunit disulfide cleavability with subsequent release of associated A chains [49], the malfunction of the DsbA could be associated with loss of fitness and perhaps virulence. A study by Peek et al. [50] showed that the periplasmic thiol:disulfide interchange protein is required for the functional maturation of secreted virulence factors of *Vibrio cholerae*.

Protease IV is a membrane endoprotease that specially hydrolyzes the signal peptide that accumulates in the cytoplasmic membrane as a result of the translocation of major lipoprotein. A protein that is to be exported across the cytoplasmic membrane of a bacterium is synthesized as a large precursor containing a signal peptide in the amino terminus (N-terminal). The signal peptide is removed from the precursor at the cytoplasmic membrane, and it is then digested by signal peptide peptidase called protease IV [51]. It is notable that the expression of protease IV is induced by quorum sensing in *P. aeruginosa* [51] and that purified protease IV was able to digest a number of biologically important proteins, including immunoglobulin, complement components, fibrinogen, and plasminogen [52]. A previous study showed that the *F. columnare* parent strain displayed the typical rhizoid colonies, while mutant exhibited smoother colony types [15]. This result, which is possibly caused by the compromised protein secretion system, is consistent with other reports that virulent isolates typically showed rhizoid colony morphology [53].

Two SNPs in the genomic area annotated as dynein protein in *F. columnare* were identified by RAST. This sequence is also present in *F. columnare* strain C#2 (Sequence ID: CP015107; query cover: 93%) and strain 94081 (Sequence ID: CP013992; query cover: 78%) by sequence comparison using BLASTn [54], but there is no annotation information under the NCBI platform. The annotation inconsistency could be due to the different annotation methods employed by RAST (i.e., Rapid Annotation using Subsystem Technology) and NCBI (i.e., Prokaryotic Genome Automatic Annotation Pipeline). Dynein proteins function as motor protein for gliding motility and intracellular cargo transport in eukaryotes, but it is not well known to present in the prokaryotes. Whether the dynein-like proteins were due to poor annotation warrants attention.

Finally, the mutation observed in the thioredoxin reductase gene might explain the decreased biofilm formation capability of Fc1723. Mutations in the thiol-oxidoreductase of *F. psychrophilum* resulted in decreased virulence and cytotoxicity, as well as enhanced biofilm formation [24]. Biofilm formation of the vaccine and parent strains was compared, and the result indicated that the mutant demonstrated both decreased virulence and biofilm formation. The phenotype of decreased biofilm could be due to the key mutation in thioredoxin reductase or to the synergistic effect of other mutations affecting protein secretion.

## 5. Conclusions

In summary, sixteen point mutations were identified in the vaccine strain. The rifampin resistance is conferred by two point mutations in the 469 and 486 aa region (Cluster I region in *rpoB*). This study identified some key mutations related to the bacterial attachment and extracellular protease secretion activity that are potential attributing regions for attenuated virulence. These results also indicated that the vaccine contains mutations in multiple genetic loci that contribute to its stability and would make reversion to a virulent phenotype unlikely. It should be noted that, although point mutations were identified, it is possible that other mutations that would be difficult to identify by the genome sequencing approach could affect phenotypes (such as gene duplications leading to increased copy numbers, and thus perhaps increased level of certain proteins).

## Figures and Tables

**Table 1:** Analysis of single nucleotide polymorphisms (SNPs) compared between vaccine strain and parent strain.

SNP Contig	SNP Position ^a^	Reference Position ^b^	SNP Pattern ^c^	Amino Acid Change ^d^	Annotation ^e^	Functional Group
scf7180000000036	125027	2071493	G > A	Asn469Lys	DNA-directed RNA polymerase beta subunit	Gene transcription
scf7180000000036	125076	2071592	G > T	His486Tyr	DNA-directed RNA polymerase beta subunit	Gene transcription
scf7180000000039	83013	1656107	C > A	Gly240stop	Nucleoside permease	Nucleosides transporter
scf7180000000044	91216	121892	G > T	Glu194Stop	Phosphoesterase	Lipid metabolism
scf7180000000039	132995	1700354	G > A	Gly386Arg	Protein-disulfide reductase	Protein biosynthesis
scf7180000000044	114715	145396	C > A	Phe577Cys	Patatin	Energy storage
scf7180000000043	500199	2767414	C > A	Ala470Asp	Protease IV	Toxin secretion
scf7180000000036	99642	2048192	T > G	Ile2902Met	Thioredoxin reductase	Redox regulation/biofilm
scf7180000000044	41423	74284	T > C	Phe238Ser	Dynein heavy chain	Unknown
scf7180000000044	41416	74277	C > T	Leu236Leu	Dynein heavy chain	Unknown
scf7180000000043	534202	2801439	C > T	Glu167Glu	Heme O synthase/protoheme IX farnesyltransferase	Protein biosynthesis
scf7180000000047	741630	1267503	G > A	NA	Hypothetical protein	NA
scf7180000000044	1361	NF	C > T	NA	Unknown region	NA
scf7180000000044	1147	NF	A > G	NA	Unknown region	NA
scf7180000000044	1363	NF	G > A	NA	Unknown region	NA
scf7180000000047	249370	804839	G > T	NA	Unknown region	NA

^a^ SNP contigs and position (in bp) are based on the FcB27 draft genome. The comparison was conducted by Mauve version 2.4, and the alignments were provided in the Table S1. ^b^ Reference position (in bp) in *F. columnare* 94081 reference genome. ^c^ SNP Pattern represents nucleotide changes. ^d^ Predicted amino acid change based on identified SNP. ^e^ Based on RAST annotation. NA, Non-Applicable. NF, Not Found.

## Data Availability

Sequence information was deposited at DDBJ/ENA/GenBank under the accession RWGX01000000 and SRKT01000000. The raw sequencing reads have been deposited in the DDBJ Sequence Read Archive under the accession number SRX5190172 and SRX5609050 for FcB27 and Fc1723, respectively. In addition, the genome sequence and annotation of FcB27 and Fc1723 were deposited in the RAST database under organism ID 996.58 and 996.57, respectively.

## Supplementary Materials

The following are available online at https://www.mdpi.com/article/10.3390/vaccines9111370/s1, Table S1, Analysis of single nucleotide polymorphisms (SNPs) compared between vaccine strain and parent strain.

## References

[1] Austin B., Austin D.A. (1999). Bacterial Fish Pathogens: Disease of Farmed and Wild Fish.

[2] USDA (2010). Part I: Reference of Catfish Health and Production Practices in the United States, 2009.

[3] Kunttu H., Sundberg L.-R., Pulkkinen K., Valtonen E.T. (2012). Environment may be the source of *Flavobacterium columanre* outbreaks at fish farms. Environ. Microbiol. Rep..

[4] Cai W., De La Fuente L., Arias C.R. (2013). Biofilm formation by the fish pathogen *Flavobacterium columnare*: Development and parameters affecting surface attachment. Appl. Environ. Microbiol..

[5] Arias C.R., LaFrentz S., Cai W., Olivares-Fuster O. (2012). Adaptive response to starvation in the fish pathogen *Flavobacterium columnare*: Cell viability and ultrastructural changes. BMC Microbiol..

[6] Tekedar H.C., Karsi A., Reddy J.S., Nho S.W., Kalindamar S., Lawrence M.L. (2017). Comparative genomics and transcriptional analysis of *Flavobacterium columnare* strain ATCC 49512. Front. Microbiol..

[7] Brudeseth B.E., Wiulsrod R., Fredriksen B.N., Kindmo K., Lokling K.-E., Bordevik M., Steine N., Klevan A., Gravningen K. (2013). Status and future perspectives of vaccines for industrialised fin-fish farming. Fish Shellfish Immunol..

[8] Woodrow K.A., Bennett K.M., Lo D.D. (2012). Mucosal vaccine design and delivery. Annu. Rev. Biomed. Eng..

[9] Shoemaker C.A., Klesius P.H., Evans J.J., Arias C.R. (2009). Use of modified live vacccine in aquaculture. J. World Aquac. Soc..

[10] Mohammed H., Olivares-Fuster O., LaFrentz S.W., Arias C.R. (2013). New attenuated vaccine against columnaris disease in fish: Choosing the right parental strain is critical for vaccine efficacy. Vaccine.

[11] Triyanto K., Wakabayashi H. (1999). Genotypic diversity of strains of *Flavobacterium columnare* from diseased fishes. Fish Pathol..

[12] LaFrentz B.R., Waldbieser G.C., Welch T.J., Shoemaker C.A. (2014). Intragenomic heterogeneity in the 16S rRNA genes of *Flavobacterium columnare* and standard protocols for genomovar assigment. J. Fish Dis..

[13] Olivares-Fuster O., Baker J.L., Terhune J.S., Shoemaker C.A., Klesius P.H., Arias C.R. (2007). Host-specific association between *Flavobacterium columnare* genomovars and fish species. Syst. Appl. Microbiol..

[14] Shoemaker C.A., Olivares-Fuster O., Arias C.R., Klesius P.H. (2008). *Flavobacterium columnare* genomovar influences mortality in channel catfish (*Ictalurus punctatus*). Vet. Microbiol..

[15] Olivares-Fuster O., Arias C.R. (2011). Development and characterization of rifampicin-resistant mutants from high virulent strains of *Flavobacterium columnare*. J. Fish Dis..

[16] Shoemaker C.A., Arias C.R., Klesius P.H., Welker T.L. (2005). Technique for identifying *Flavobacterium columnare* using whole-cell fatty acid profiles. J. Aquat. Anim. Health.

[17] Wei Z.-G., Zhang S.-W. (2018). NPBSS: A new PacBio sequencing simulator for generating the continuous long reads with an empirical model. BMC Bioinform..

[18] Overbeek R., Olson R., Pusch G.D., Olsen G.J., Davis J.J., Disz T., Edwards R.A., Gerdes S., Parrello B., Shukla M. (2013). The SEED and the Rapid Annotation of microbial genomes using Subsystems Technology (RAST). Nucleic Acids Res..

[19] Aziz R.K., Bartels D., Best A.A., DeJongh M., Disz T., Edwards R.A., Formsma K., Gerdes S., Glass E.M., Kubal M. (2008). The RAST Server: Rapid annotations using subsystems technology. BMC Genom..

[20] Darling A.C., Mau B., Blattner F.R., Perna N.T. (2004). Mauve: Multiple alignment of conserved genomic sequence with rearrangements. Genome Res..

[21] Thorvaldsdóttir H., Robinson J.T., Mesirov J.P. (2013). Integrative Genomics Viewer (IGV): High-performance genomics data visualization and exploration. Brief. Bioinform..

[22] Choi Y., Chan A.P. (2015). PROVEAN web server: A tool to predict the functional effect of amino acid substitutions and indels. Bioinformatics.

[23] Goldstein B.P. (2014). Resistance to rifampicin: A review. J. Antibiot..

[24] Alvarez B., Secades P., Prieto M., McBride M.J., Guijarro J.A. (2006). A mutation in *Flavobacterium psychrophilum* tlpB inhibits gliding motility and induce biofilm formation. Appl. Environ. Microbiol..

[25] Minor P.D. (2015). Live attenuated vaccines: Historical successes and current challenges. Virology.

[26] Shoemaker C.A., Klesius P.H. (1997). Protective immunity against enteric septicaemia in channel catfish, *Ictalurus punctatus* (Rafinesque), following controlled exposure to *Edwardsiella ictaluri*. J. Fish Dis..

[27] Thune R.L., Fernandez D.H., Battista J.R. (1999). An aroA Mutant of *Edwardsiella ictaluri* is Safe and Efficacious as a Live, Attenuated Vaccine. J. Aquat. Anim. Health.

[28] Detmer A., Glenting J. (2006). Live bacterial vaccines—A review and identification of potential hazards. Microb. Cell Fact..

[29] Montaraz J.A., Winter A.J. (1986). Comparison of living and nonliving vaccines for Brucella abortus in BALB/c mice. Infect. Immun..

[30] Dietrich G., Collioud A., Rothen S.A. (2008). Developing and manufacturing attenuated live bacterial vaccines. BioPharm Int..

[31] Gliniewicz K., Wildung M., Orfe L.H., Wiens G.D., Cain K.D., Lahmers K.K., Snekvik K.R., Call D.R. (2015). Potential mechanisms of attenuation for rifampicin-passaged strains of *Flavobacterium psychrophilum*. BMC Microbiol..

[32] Schurig G.G., Roop R.M., Bagchi T., Boyle S., Buhrman D., Sriranganathan N. (1991). Biological properties of RB51; a stable rough strain of *Brucella abortus*. Vet. Microbiol..

[33] LaFrentz B.R., LaPatra S.E., Call D.R., Cain K.D. (2008). Isolation of rifampicin resistant *Flavobacterium psychrophilum* strains and their potential as live attenuated vaccine candidates. Vaccine.

[34] Klesius P.H., Shoemaker C.A. (1999). Development and use of modified live *Edwardsiella ictaluri* vaccine against enteric septicemia of catfish. Adv. Vet. Med..

[35] Shoemaker C.A., Klesius P.H., Drennan J.D., Evans J.J. (2011). Efficacy of a modified live *Flavobacterium columnare* vaccine in fish. Fish Shellfish Immunol..

[36] Wehrli W., Knüsel F., Schmid K., Staehelin M. (1968). Interaction of rifamycin with bacterial RNA polymerase. Proc. Natl. Acad. Sci. USA.

[37] Ovchinnikov Y.A., Monastyrskaya G.S., Guriev S.O., Kalinina N.F., Sverdlov E.D., Gragerov A.I., Bass I.A., Kiver I.F., Moiseyeva E.P., Igumnov V.N. (1983). RNA polymerase rifampicin-resistance mutations in *Escherichia coli*: Sequence changes and dominance. Mol. Gen. Genet..

[38] Jin D.J., Gross C.A. (1988). Mapping and sequencing of mutations in the *Escherichia coli* rpoB gene that lead to rifampicin resistance. J. Mol. Biol..

[39] Arias C.R., Shoemaker C.A., Evans J.J., Klesius P.H. (2003). A comparative study of *Edwardsiella ictaluri* parent (EILO) and *E. ictaluri* rifampicin-mutant (RE-33) isolates using lipopolysaccharides, outer membrane proteins, fatty acids, Biolog, API 20E and genomic analyses. J. Dis..

[40] Vemulapalli R., Mc Quiston J.R., Schurig G.G., Sriranganathan N., Halling S.M., Boyle S.M. (1999). Identification of an IS711 element interrupting the wboA Gene of *Brucella abortus* vaccine Strain RB51 and a PCR Assay to distinguish strain RB51 from other *Brucella* species and strains. Clin. Diagn. Lab. Immunol..

[41] Weissman S.J., Moseley S.L., Dykhuizen D.E., Sokurenko E.V. (2003). Enterobacterial adhesins and the case for studying SNPs in bacteria. Trends Microbiol..

[42] de Koning H., Diallinas G. (2000). Nucleobase transporters. Mol. Membr. Biol..

[43] Vogels G.V.D., Van der Drift C. (1976). Degradation of purines and pyrimidines by microorganisms. Bacteriol. Rev..

[44] Smith S.W., Weiss S.B., Kennedy E.P. (1957). The enzymatic dephosphorylation of phosphatidic acids. J. Biol. Chem..

[45] Andrews D., Beames B., Summers M., Park W. (1988). Characterization of the lipid acyl hydrolase activity of the major potato (*Solanum tuberosum*) tuber protein, patatin, by cloning and abundant expression in a *baculovirus* vector. Biochem. J..

[46] Sato H., Frank D.W., Hillard C.J., Feix J.B., Pankhaniya R.R., Moriyama K., Finck-Barbançon V., Buchaklian A., Lei M., Long R.M. (2003). The mechanism of action of the *Pseudomonas aeruginosa*-encoded type III cytotoxin, *ExoU*. EMBO J..

[47] Phillips R.M., Six D.A., Dennis E.A., Ghosh P. (2003). In vivo phospholipase activity of the *Pseudomonas aeruginosa* cytotoxin *ExoU* and protection of mammalian cells with phospholipase A2 inhibitors. J. Biol. Chem..

[48] Banerji S., Flieger A. (2004). Patatin-like proteins: A new family of lipolytic enzymes present in bacteria?. Microbiology.

[49] Hermanson G.T. (2013). Bioconjugate Techniques.

[50] Peek J.A., Taylor R.K. (1992). Characterization of a periplasmic thiol: Disulfide interchange protein required for the functional maturation of secreted virulence factors of *Vibrio cholerae*. Proc. Natl. Acad. Sci. USA.

[51] Suzuki T., Itoh A., Ichihara S., Mizushima S. (1987). Characterization of the sppA gene coding for protease IV, a signal peptide peptidase of *Escherichia coli*. J. Bacteriol..

[52] Engel L.S., Hill J.M., Caballero A.R., Green L.C., O’Callaghan R.J. (1998). Protease IV, a unique extracellular protease and virulence factor from *Pseudomonas aeruginosa*. J. Biol. Chem..

[53] Kunttu H., Jokinen E., Valtonen E., Sundberg L.R. (2011). Virulent and nonvirulent *Flavobacterium columnare* colony morphologies: Characterization of chondroitin AC lyase activity and adhesion to polystyrene. J. Appl. Microbiol..

[54] Mount D.W. (2007). Using the basic local alignment search tool (BLAST). Cold Spring Harb. Protoc..

