# Supplementary material for: Deciphering the Molecular Basis for Attenuation of Flavobacterium columnare Strain Fc1723 Used as Modified Live Vaccine against Columnaris Disease
Source: Vaccines (Basel). 2021 Nov 22;9(11):1370. doi: 10.3390/vaccines9111370 (PMC8622145; doi:10.3390/vaccines9111370)
Supplement: Supplementary file 1 [file vaccines-09-01370-s001.zip › vaccines-1457960-supplementary.pdf]

Supplementary Table S1.

Table S1. Analysis of single nucleotide polymorphisms (SNPs) compared between vaccine strain and parent strain.

SNP_Pattern	FcB27_Contig	FcB27_PosInContig	Fc1723_Contig	Fc1723_PosInContig	RAST Annotation ID
TG	scf7180000000036	99642	scf7180000000021	520299	fig|996.58.peg.158
GA	scf7180000000036	125027	scf7180000000021	545685	fig|996.58.peg.168
GT	scf7180000000036	125076	scf7180000000021	545734	fig|996.58.peg.168
CA	scf7180000000039	83013	scf7180000000021	98057	fig|996.58.peg.405
GA	scf7180000000039	132995	scf7180000000021	148039	fig|996.58.peg.443
CA	scf7180000000043	500199	scf7180000000023	1492283	fig|996.58.peg.1308
CT	scf7180000000043	534202	scf7180000000023	1526286	fig|996.58.peg.1341
GT	scf7180000000044	91216	scf7180000000018	340971	fig|996.58.peg.1428
TC	scf7180000000044	41423	scf7180000000018	390758	fig|996.58.peg.1396
CT	scf7180000000044	41416	scf7180000000018	390765	fig|996.58.peg.1396
GA	scf7180000000044	1363	scf7180000000018	430889	none CDS or RNA
CT	scf7180000000044	1361	scf7180000000018	430893	none CDS or RNA
AG	scf7180000000044	1147	scf7180000000018	431113	none CDS or RNA
GA	scf7180000000047	741630	scf7180000000023	245486	hypothetical protein
GT	scf7180000000047	249370	scf7180000000023	737762	none CDS or RNA
CA	scf7180000000044	114715	scf7180000000018	317483	none CDS or RNA
